# Broadband Acoustic Purcell Effect from Collective Bound States in the Continuum

**DOI:** 10.1002/advs.202414627

**Published:** 2025-02-21

**Authors:** Sibo Huang, Shuhuan Xie, Tuo Liu, Tong Hao, Din Ping Tsai, Yong Li, Jie Zhu

**Affiliations:** ^1^ Department of Electrical Engineering City University of Hong Kong Hong Kong SAR 999077 China; ^2^ Institute of Acoustics Tongji University Shanghai 200092 China; ^3^ Key Laboratory of Noise and Vibration Research, Institute of Acoustics Chinese Academy of Sciences Beijing 100190 China; ^4^ College of Surveying and Geo‐Informatics Tongji University Shanghai 200092 China

**Keywords:** bound states in the continuum, broadband acoustic Purcell effect, collective mode responses, emission‐enhancement materials, symmetry‐related modulation

## Abstract

The Purcell effect significantly improves the performance of various emission devices but is typically constrained by a narrow operational bandwidth due to inherent resonant mechanisms. This study achieves broadband acoustic Purcell effect, substantially boosting sound emission by exploring collective quasibound states in the continuum (QBICs). A six‐cavity coupled system supporting five QBICs is introduced, wherein all of the QBICs interact strongly with an acoustic source. This system takes advantage of the high quality factors and the strong mode responses of the collective QBICs, leading to a substantial enhancement of the local density of states. Consequently, a considerable increase in sound emission is realized across the frequency range of 625–900 Hz. These findings provide insights into the physical mechanisms driving the broadband Purcell effect in resonant systems and open up promising avenues for the development of advanced acoustic emission devices.

## Graphical abstract

This study introduces a novel six‐cavity system that utilizes collective quasibound states in the continuum (QBICs) to achieve a broadband acoustic Purcell effect. By strongly coupling five QBICs with an acoustic source within the introduced system, the research demonstrates a significant enhancement in sound emission from 625 to 900 Hz, paving the way for developing advanced acoustic emission devices.

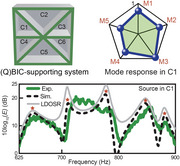

## 1 Introduction

Purcell's seminal work in 1946 indicated that the spontaneous emission rate of an emitter can be altered by its surrounding environment,^[^ ^1^ ^]^ a phenomenon known as the Purcell effect. This effect has become instrumental in modulating emission properties across diverse domains of wave physics.^[^ ^2^, ^3^, ^4^, ^5^, ^6^ ^]^ Bound states in the continuum (BICs)^[^ ^7^, ^8^, ^9^, ^10^, ^11^, ^12^, ^13^, ^14^, ^15^ ^]^ demonstrate remarkable potential for the strong Purcell effect. BICs are completely confined states within a continuous radiating‐wave spectrum, which exhibit exceptional physical characteristics such as infinitely large quality factors and enhanced wave‐matter interactions. Transitioning BICs into quasibound states in the continuum (QBICs) provides access to external radiation while preserving large quality factors, thus enabling QBIC‐based applications with high intensity and sensitivity, such as lasing,^[^ ^16^, ^17^, ^18^ ^]^ sensing,^[^ ^19^, ^20^, ^21^, ^22^, ^23^, ^24^, ^25^ ^]^ absorption,^[^ ^26^, ^27^, ^28^, ^29^ ^]^ and nonlinearity enhancement.^[^ ^30^, ^31^, ^32^, ^33^ ^]^ The strong Purcell effect requires high quality factors. Therefore, by leveraging a QBIC, the strong Purcell effect for emission enhancement in a relatively narrow frequency band can be achieved.^[^ ^34^, ^35^, ^36^, ^37^, ^38^, ^39^ ^]^ However, a high quality factor is typically associated with a narrow operational bandwidth. As a result, achieving both strong and broadband Purcell effect poses a significant challenge due to this inherent trade‐off.^[^ ^40^, ^41^, ^42^, ^43^, ^44^, ^45^ ^]^

In this study, we achieve broadband Purcell effect for emission enhancement by exploiting collective QBICs. Modulating emission through collective modes is more complex than modulating absorption, reflection, or transmission. In the latter scenarios, broadband performance can be facilitated when each mode achieves a strong response in any spatial domain of the resonant system. However, in emission scenarios involving a specific wave source, it is essential for all collective modes to achieve strong responses at the same spatial domain to enable effective interactions with the source, thus presenting a considerable challenge. To address this, we conduct an in‐depth theoretical analysis of the underlying mechanisms governing the formation of collective QBICs supported by a multi‐resonator system. Building on this foundation, we develop a strategic approach to effectively manipulate their mode responses, enabling strong mode responses at the acoustic source for all target QBICs. Our research explores the intricate interplay between the broadband acoustic Purcell effect and collective QBICs, paving the way for unprecedented control over emission in acoustic devices, thereby enhancing their efficiency and bandwidth.

## 2 Results and Discussion

### 2.1 Concept and Theoretical Analysis

The Purcell effect is fundamentally related to the local density of states (LDOS, *ξ*) within resonant systems. The LDOS has a linear positive correlation with the emitted power *E*_p_ of an acoustic source, given by $E_{p}=\pi \rho_{0}c_{0}^{2}{\left|A_{s}\right|}^{2}\xi$, where *ρ*_0_ represents the density of air, *c*_0_ indicates the sound speed in air, *A*_s_ denotes the source strength. Therefore, the LDOS serves as an effective tool for theoretically evaluating the Purcell effect without intrinsic (thermal‐viscous) loss. The LDOS at the source location $\vec{r_{0}}$ can be expressed as^[^ ^46^ ^]^

(1) ξ(ω,r⃗0)=−dk02(ω)dω1πIm[G(ω,r⃗=r⃗0,r⃗0)]

where $k_{0}^{2}=\omega^{2}/c_{0}^{2}$, Im[*G*] represents the imaginary part of the Green's function. By utilizing the quasinormal mode (QNM) theory^[^ ^47^, ^48^, ^49^ ^]^ in coupled open resonant systems, the Green's function at the source location ${\vec{r}}_{0}$ can be expressed as

(2) $$G\left(\omega ,\vec{r},{\vec{r}}_{0}\right)=\sum_{n}\left[\frac{{c_{0}}^{2}}{\left(\omega_{n}+i\gamma_{n}\right)\left(\omega_{n}+i\gamma_{n}-\omega \right)}\frac{p\left(\vec{r}\right)p\left({\vec{r}}_{0}\right)}{\int p^{2}\left(\vec{r}\right)dV}\right]$$

where *ω*_*n*_ and *γ*_*n*_ indicate the resonant angular frequency and the radiative decay rate of the *n*‐th mode, *ω* is the angular frequency, $p(\vec{r})$ and $p({\vec{r}}_{0})$ represent the pressures amplitudes at the locations of $\vec{r}$ and ${\vec{r}}_{0}$, $\int p^{2}\left(\vec{r}\right)dV$ indicates the integration of $p^{2}\left(\vec{r}\right)$ in the overall system. By substituting Equation (2) into Equation (1), we obtain

(3) $$\xi \left(\omega ,{\vec{r}}_{0}\right)=\frac{1}{\pi }\sum_{n}\left\{\frac{p^{2}\left({\vec{r}}_{0}\right)}{V_{m}\max\left(p^{2}\left(\vec{r}\right)\right)}\frac{1}{\gamma_{n}}\right\}$$

where *V*_m_ indicates the model volume of the system with $V_{m}=\frac{\int p^{2}\left(\vec{r}\right)dV}{\max(p^{2}\left(\vec{r}\right))}$, $\max(p^{2}\left(\vec{r}\right))$ represents the maximum value of $p^{2}\left(\vec{r}\right)$ in the system. For the systems consisting of acoustic Fabry–Pérot resonators, *V*_m_ approximates the total spatial volume of these systems.^[^ ^50^ ^]^ Equation (3) manifests that a smaller radiative decay rate *γ*_*n*_ leads to a larger LDOS at resonance, thereby facilitating the stronger Purcell effect for reinforced emission. QBICs are particularly promising for enhancing the Purcell effect due to their typically low *γ* values, ranging from infinitesimally small to moderately small.^[^ ^51^, ^52^, ^53^, ^54^, ^55^ ^]^ In practical acoustic systems, the optimal quality factors of QBICs are determined by the balance between radiation and intrinsic losses.^[^ ^56^ ^]^ Besides, the mode response factor is defined as $M_{r}=\frac{p({\vec{r}}_{0})}{\max(p\left(\vec{r}\right))}$, which plays a crucial role in influencing the LDOS and emission properties, as indicated by Equation (3). As a result, high quality factors and strong mode responses mutually promote the strong acoustic Purcell effect.

**Figure 1:**
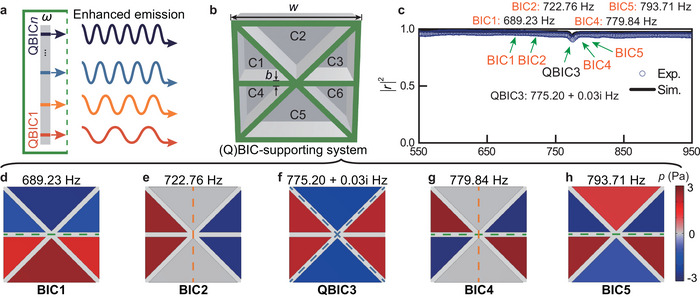
Concept and implementation of the QBIC‐induced broadband Purcell effect. a) An acoustic system supporting collective QBICs leads to broadband emission enhancement. The arrows with different colors indicate QBICs at different frequencies. b) Schematic of the six‐cavity coupled system. The wall thickness (*b*) is 5 mm. c) Reflection spectrum of the structure in b). Exp., experiment. Sim., simulation. d–h) Pressure eigenfields of the five (Q)BICs (structure's bottom view) and their eigenfrequencies. The colored dashed lines indicate the symmetric lines of the (Q)BICs, where the pressure of a complete mode varies from the minimum value to the maximum value.

Furthermore, given the inherent contradiction between the strength and bandwidth of the Purcell effect observed in single resonant modes, we strategically employ collective QBICs **Figure** **1**a. While this approach introduces several complex challenges (to be demonstrated later), it allows us to bypass the inherent contradiction and opens promising avenues for achieving broadband Purcell effect upon the successful resolution of these introduced challenges. First, a six‐cavity coupled system is proposed, which supports four pure BICs and one QBIC when all the acoustic Fabry‐Pérot cavities have the same depth (100 mm) Figure 1b. The depth of the cavities was selected to ensure that the fundamental order of reflection is dominant.^[^ ^57^, ^58^, ^59^, ^60^, ^61^ ^]^ Other suitable depth values (such as 150 or 200 mm) can also achieve similar (Q)BICs at different frequencies. By appropriately modifying the symmetry properties of the system, five QBICs can be achieved. The presence of BICs and QBICs is experimentally demonstrated by the vanishing linewidth phenomena^[^ ^17^, ^27^ ^]^ in the systems' reflection spectra Figure 1c (see more information in Figures S1–S3, Supporting Information), and theoretically proved by the eigenvalue evolution analysis (see Figure S4, Supporting Information). The cross‐section of the overall six‐cavity coupled system is consistent with the cross‐sectional size of the experimental square waveguide having a width (*w*) of 100 mm. Since the intrinsic loss is associated with the narrowness of the cavities' cross‐sections, the selected number of cavities (six) is chosen to achieve the appropriate intrinsic loss. The configuration of triangular cavities is designed to make the cavities close to each other, enabling strong inter‐cavity coupling. Cavities C1, C3, C4, and C6 have the same cross‐section, and cavities C2 and C5 have the same cross‐section but are different from the other four cavities. The different cross‐sectional sizes of the cavities can introduce varied radiation and near‐field couplings, which reduces the degeneracy of the system modes, forms different symmetry classes, and allows the (Q)BICs to distribute over a broader frequency range more effectively. By adjusting cavity depths, the BICs can turn into QBICs. If a single target cavity is tuned to exhibit strong mode responses to all five QBICs, the acoustic source within the target cavity can achieve broadband emission enhancement (see schematic illustration in Figure S5, Supporting Information).

### 2.2 Theoretical Analysis of the BICs and QBICs Supported by the Six‐Cavity Coupled System

The fundamental characteristics of a resonant mode can be represented by its eigenvalue ((*ω*_0_ + *i* · *γ*_0_)/(2π)), where *ω*_0_ corresponds to the resonant angular frequency and *γ*_0_ corresponds to the radiative decay rate. *γ*_0_ = 0 indicates that the mode does not radiate to the far field. The six‐cavity coupled system presented in this study exemplifies a typical waveguide‐resonator system, wherein the supported BICs are formed through the mechanism of coupled resonances.^[^ ^7^ ^]^ To elucidate the formation of these BICs, mode analysis focusing on the system's eigenvalues is a common and effective approach.^[^ ^8^ ^]^ The investigated modes supported by our presented systems exist within the continuum spectrum of radiating waves, and thus these modes with *γ*_0_ = 0 are classified as BICs. Figure 1d–h demonstrates the eigenfields and eigenvalues of the five (Q)BICs based on simulations with COMSOL Multiphysics, intuitively demonstrating the symmetry properties of the (Q)BICs and the mode responses of the six cavities. For QBIC3, we will demonstrate later that it evolves from a pure BIC, hence it is classified as a QBIC. The colored dashed lines indicate the symmetric lines of the (Q)BICs, where the pressure of a complete mode varies from the minimum value to the maximum value. Besides, the eigenfield distributions of five (Q)BICs can be classified into the irreducible representations of the *C*_2*v*_ group.^[^ ^62^ ^]^ Specifically, BIC1 corresponds to the *B*_2_ irreducible representation; BIC2 corresponds to the *B*_1_ irreducible representation; QBIC3 corresponds to the *A*_1_ irreducible representation; BIC4 corresponds to the *A*_2_ irreducible representation; BIC5 corresponds to the *B*_2_ irreducible representation (see Table S1 and Figure S6, Supporting Information). The pressure fields are shown from the bottom view of the system. Additionally, the 3D and amplitude illustrations of the pressure fields are provided in Figures S7–S10, Supporting Information, demonstrating that the maximum pressure amplitudes occur at the bottoms of the cavities. Therefore, we will later set the acoustic source at the bottom centers of the cavities to achieve strong mode responses. Based on the symmetry properties, the couplings among the cavities can be categorized into six types (*χ*_1_‐*χ*_6_, see Figure S11, Supporting Information). Employing the temporal coupled‐mode theory,^[^ ^26^, ^63^, ^64^ ^]^ the Hamiltonian matrix for the presented six‐cavity coupled system can be expressed as

(4) $$H=\left(\begin{array}{cccccc} \Psi_{1} & \chi_{1} & \chi_{2} & \chi_{3} & \chi_{4} & \chi_{5}\\ \chi_{1} & \Psi_{2} & \chi_{1} & \chi_{4} & \chi_{6} & \chi_{4}\\ \chi_{2} & \chi_{1} & \Psi_{3} & \chi_{5} & \chi_{4} & \chi_{3}\\ \chi_{3} & \chi_{4} & \chi_{5} & \Psi_{4} & \chi_{1} & \chi_{2}\\ \chi_{4} & \chi_{6} & \chi_{4} & \chi_{1} & \Psi_{5} & \chi_{1}\\ \chi_{5} & \chi_{4} & \chi_{3} & \chi_{2} & \chi_{1} & \Psi_{6} \end{array}\right)$$

where Ψ_(*n*)_ = *ω*_(*n*)_ + *i* *γ*_(*n*)_. *ω*_(*n*)_ and *γ*_(*n*)_ indicate the resonant angular frequency and the radiative decay rate of cavity C(*n*), respectively. *χ*_(*j*)_ = *κ*_(*j*)_ + *i* *γ*_fc(*j*)_. *κ*_(*j*)_ and *i* *γ*_fc(*j*)_ represent the near‐field coupling factor and the radiation coupling factor between the two cavities corresponding to coupling type *j*, respectively; *n*, *j* = 1, 2, …, 6. Given the subwavelength distances among the cavities, we can assume $\gamma_{\mathrm{fc}1}=\gamma_{\mathrm{fc}4}=\sqrt{\gamma_{1}\gamma_{2}}$, *γ*_fc2_ = *γ*_fc3_ = *γ*_fc5_ = *γ*_1_, and *γ*_fc6_ = *γ*_2_. Then, the six eigenvalues of the Hamiltonian matrix can be deduced as shown in **Table** **1**, where *ζ* = (8(*χ*_1_ − *χ*_4_)^2^ + (*χ*_2_ + *χ*_6_ − *χ*_3_ − *χ*_5_ + *ω*_1_ − *ω*_2_ + *i*(*γ*_1_ − *γ*_2_))^2^)^1/2^ and *ζ*′ = (8(*χ*_1_ + *χ*_4_)^2^ + (*χ*_2_ + *χ*_3_ + *χ*_5_ − *χ*_6_ + *ω*_1_ − *ω*_2_ + *i*(*γ*_1_ − *γ*_2_))^2^)^1/2^.

**Table 1:** Eigenvalues of the Hamiltonian matrix in Equation (4).

No.	Eigenvalues
*σ*_1_	*χ*_3_ − *χ*_2_ − *χ*_5_ + *ω*_1_ + *i* *γ*_1_
*σ*_2_	*χ*_5_ − *χ*_2_ − *χ*_3_ + *ω*_1_ + *i* *γ*_1_
*σ*_3_	$\frac{1}{2}\left(\chi_{2}-\chi_{3}-\chi_{5}-\chi_{6}+\omega_{1}+\omega_{2}+i\left(\gamma_{1}+\gamma_{2}\right)-\zeta \right)$
*σ*_4_	$\frac{1}{2}\left(\chi_{2}-\chi_{3}-\chi_{5}-\chi_{6}+\omega_{1}+\omega_{2}+i\left(\gamma_{1}+\gamma_{2}\right)+\zeta \right)$
*σ*_5_	$\frac{1}{2}\left(\chi_{2}+\chi_{3}+\chi_{5}+\chi_{6}+\omega_{1}+\omega_{2}+i\left(\gamma_{1}+\gamma_{2}\right)-\zeta^{\prime }\right)$
*σ*_6_	$\frac{1}{2}\left(\chi_{2}+\chi_{3}+\chi_{5}+\chi_{6}+\omega_{1}+\omega_{2}+i\left(\gamma_{1}+\gamma_{2}\right)+\zeta^{\prime }\right)$

**Figure 2:**
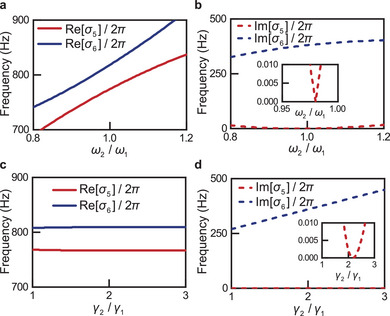
Theoretical analysis of (Q)BIC3. a,b) Numerical demonstrations of *σ*_5_ and *σ*_6_. The real parts (Re) and imaginary parts (Im) of *σ*_5_ and *σ*_6_ with varying ratios between *ω*_2_ and *ω*_1_, while keeping *ω*_1_= (762 × 2π) Hz, *γ*_1_ = 0.059*ω*_1_, *γ*_2_ = 0.132*ω*_2_, $\kappa_{1}=0.0305\sqrt{\omega_{1}\omega_{2}}$, *κ*_2_ = 0.0163*ω*_1_, *κ*_3_ = −0.0105*ω*_1_, $\kappa_{4}=-0.0103\sqrt{\omega_{1}\omega_{2}}$, *κ*_5_ = 0.0271*ω*_1_, *κ*_6_ = 0.0568*ω*_2_. The fixed parameters are based on the structure in Figure 1 (see details in Section F, Supporting Information). c,d) The real parts and imaginary parts of *σ*_5_ and *σ*_6_ with varying ratios between *γ*_2_ and *γ*_1_, while setting *ω*_1_= (762 × 2π) Hz, *ω*_2_= (746.6 × 2π) Hz, *γ*_1_ = 0.059*ω*_1_, with the rest parameters the same as in (a,b). See more information on (b) and (d) in Figure S12 (Supporting Information).

After substituting the expressions of *χ*_1_‐*χ*_6_ into the six eigenvalues and conducting mathematical deductions, the first four eigenvalues lead to real numbers, corresponding to the four BICs demonstrated in Figure 1. Furthermore, although the expressions of *σ*_5_ and *σ*_6_ are relatively complex, numerical calculations manifest that *σ*_5_ can turn into a BIC when the ratio between *ω*_1_ and *ω*_2_ reaches a specific value ≈1 **Figure** **2**a,b. For other ratios between *ω*_1_ and *ω*_2_, the imaginary part of *σ*_5_ remains relatively small, which corresponds the condition in Figure 1f. Therefore, the mode shown in Figure 1f is a QBIC since it evolves from a pure BIC. Conversely, the imaginary part of *σ*_6_ is notably large, corresponding to a lossy mode. Furthermore, similar results can be obtained by varying other parameters, such as *γ*_2_/*γ*_1_ Figure 2c,d.

**Figure 3:**
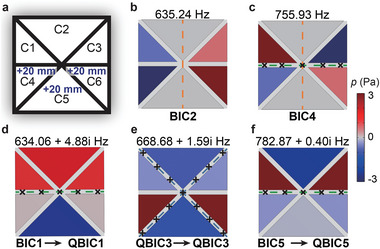
Turning BICs into QBICs. a) Modulation of the cavities' depths. b,c) BIC2 and BIC4 maintain pure BICs. d–f) BIC1 and BIC5 turn into QBICs.

**Figure 4:**
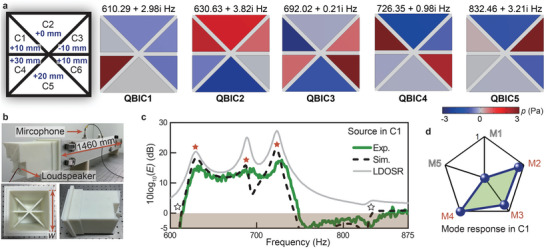
Emission enhancement induced by collective QBICs. a) Modulation of cavity depths and the five QBICs. b) Experimental setup and sample. c) Measured (green line) and simulated (black dashed line) Purcell factors (*E*) with the acoustic source in cavity C1, along with LDOSR at the acoustic source (grey line). The yellow‐filled and hollow pentagrams mark the QBICs capable of and incapable of inducing significant emission enhancement, respectively. d) The mode responses to the five QBICs for the acoustic source in cavity C1. M1‐M5 correspond to QBIC1‐QBIC5 and the axis is logarithmically ranging from 0.1 to 1.

Pure BICs are isolated modes that defy radiation, thus incapable of inducing emission phenomena. However, QBICs maintain high quality factors and meanwhile allow external radiation. Therefore, we deliberately disrupt the symmetry properties of the six‐cavity coupled system to turn these BICs into QBICs. Among the four BICs, three of them (BIC1, BIC4, and BIC5) are associated with the transverse symmetric lines of mode construction, and thus we first demonstrate the effect of breaking the transverse symmetries by increasing the depth of cavities C4–C6 by 20 mm **Figure** **3**a. As shown in Figure 3b–f, BIC1 and BIC5 turn into QBICs since all of their reliant symmetries are broken. However, the preserved vertical symmetries allow BIC2 and BIC4 to maintain pure BICs. Subsequently, we further introduce cavities' depth differences to transform BIC2 and BIC4 into QBICs for the potential of the broadband Purcell effect. This adjustment involves increasing the depths of cavity C1 and cavity C4 by 10 mm, while decreasing the depths of cavity C3 and cavity C6 by 10 mm on the basis of the structure in Figure 3a. As a result, all of the five (Q)BICs successfully turn into QBICs with eigenfrequencies' imaginary parts **Figure** **4**a. Note that the modulation approach demonstrated above represents one of many possible methods for showcasing the system's symmetry properties.

### 2.3 Implementation of the QBIC‐Induced Broadband Purcell Effect

The Purcell factor defined by the emission ratio of *E* = |*P*_T_/*P*_T0_|^2^ is utilized to evaluate the Purcell effect, where *P*_T_ and *P*_T0_ represent the amplitudes of emitted waves with the presented structure and with an empty tube, respectively.^[^ ^39^, ^46^, ^65^, ^66^ ^]^ Besides, the LDOS ratio (LDOSR), denoted as *ξ*_a_/*ξ*_0_, can predict the emission enhancement of acoustic sources in the absence of intrinsic loss, where *ξ*_a_ and *ξ*_0_ indicate the LDOS at the acoustic source in the QBIC‐supporting system and in the empty tube, respectively.^[^ ^66^ ^]^ Due to the intrinsic loss in practical acoustic systems, the measured Purcell factors will be lower than the LDOSR.^[^ ^39^, ^46^ ^]^ To experimentally investigate the emission properties, we conduct measurements in a steel waveguide with a length of 1460 mm, a square cross‐section with a side length of 100 mm, and a wall thickness of 8 mm Figure 4b.

The eigenmodes of the five QBICs Figure 4a suggest that this structure has the potential for achieving emission enhancement from 610 to 832 Hz, provided all five QBICs effectively interact with the acoustic source. However, the practical working frequency bandwidth is significantly narrower than anticipated. Specifically, as shown in Figure 4c, the experimental results of 10log_10_(*E*) consistently exceed 10 dB in the frequency range from 623 to 740 Hz, with a peak of 17.6 dB at 729 Hz. Nevertheless, overall emission enhancement is observed only from 613 to 748 Hz. The experimentally demonstrated Purcell effect is valid for common membrane‐vibration‐based loudspeakers (see detailed discussions in Section H, Supporting Information). In addition, the directly measured sound pressure with structures and the empty tube is provided in Figure S13 (Supporting Information). From Figure 4c, it can be observed that when the acoustic source is placed at the bottom center of cavity C1, only QBIC2, QBIC3, and QBIC4 induce notable emission enhancement, whereas QBIC1 and QBIC5 fail to be excited as evidenced by the spectrum of Purcell factors.

The underlying mechanism causing the unsatisfactory results in Figure 4c is the weak mode responses ($M_{r}=\frac{p({\vec{r}}_{0})}{\max(p\left(\vec{r}\right))}$). Here, $p({\vec{r}}_{0})$ represents the pressure amplitude at the acoustic source and $\max(p(\vec{r})$) indicates the maximum pressure amplitude inside the entire system with respect to the pressure distributions of the (Q)BIC eigenmodes. Therefore, the maximum value of the mode response factor is 1, which can be realized when the maximum pressure amplitude in the QBIC‐supporting system occurs at the location of the acoustic source. The calculated mode responses to the five QBICs at the acoustic source in cavity C1 uncover that the strong mode responses are achieved at QBIC2, QBIC3, and QBIC4, corresponding to the frequency ranges with remarkable sound emission enhancement. Nevertheless, weak mode responses are observed at QBIC1 and QBIC5, corresponding to the frequency ranges with low or even restricted (*E* < 1) emission Figure 4d. Here, we regard *M*_r_ > 0.4 as the threshold of strong mode response. Additionally, it can be observed that the emission of the source is restricted in some frequency bands such as within 760–830 Hz Figure 4c. This phenomenon suggests potential applications in noise control engineering for reducing the emission of noise sources. In this study, our primary focus is the achievement of broadband emission enhancement and this emission restriction phenomenon should be avoided within the target frequency band. It is also noteworthy that the presence of multiple sources can disrupt the QBICs supported by the system. Therefore, employing a single source in the presented system is a more effective approach for the broadband Purcell effect (see detailed explanations in Section J, Supporting Information).

Achieving simultaneous strong mode responses to multiple modes for a single resonator within a multi‐resonator system represents a considerable obstacle. For the structure shown in Figure 4d, the remaining five cavities are also unable to support strong mode responses to all five QBICs, typically limited to 1‐2 strong mode responses to the QBICs for each cavity (see Figure S14, Supporting Information). This arouses an intriguing and fresh question: How can a single resonator in a multi‐resonator system simultaneously achieve strong mode responses to collective modes (QBICs)? This question bears a resemblance to the studies regarding the “rainbow trapping” effect^[^ ^67^, ^68^ ^]^ and coupled‐resonant broadband sound absorption,^[^ ^69^, ^70^, ^71^ ^]^ where strong responses to multiple modes are sought. However, in structures promoting rainbow trapping and coupled broadband absorbers with multiple component resonators, each resonator typically exhibits strong mode responses to only a small part of the target modes, commonly one or two.^[^ ^66^, ^72^ ^]^ However, in the scenario of the broadband Purcell effect, a single cavity must possess strong responses to a relatively large number of QBICs, rendering the question more complicated and challenging. In the following, we will show how this question can be effectively addressed through the engineered symmetry properties of the six‐cavity coupled system.

The five QBICs are all constructed with significant mutual interactions among multiple resonators, leading to strong nonlocality features^[^ ^73^ ^]^ as well as complex mode responses of these QBICs. This means that modulation of a single resonator within the multi‐resonator system can lead to simultaneous changes in multiple resonators' mode responses across various modes. Consequently, it is challenging to modulate the collective QBICs' mode responses of a single resonator independently, which therefore hinders the realization of strong mode responses to all five QBICs for one target resonator. Employing optimization algorithms and machine learning strategies together with numerical calculations could facilitate the modulation of mode responses. However, in this work, we present a symmetry‐modulation strategy. Specifically, for each QBIC, at least one cavity must exhibit the strong mode response, as the eigenfield's largest pressure amplitudes are inherently distributed within the six‐cavity coupled system. Therefore, it is possible to reinforce the mode responses of a selected cavity by weakening the mode responses of other cavities. In this study, we weaken the mode responses of cavities C2 and C5, given their inferior contributions to the original BIC2 and BIC4 Figure 1d–h. The constructions of the five QBICs are relevant to the symmetry properties of the cavities, and thus we can weaken or enhance the mode responses of specific cavities by impairing or improving their associated symmetries in the QBIC constructions.

**Figure 5:**
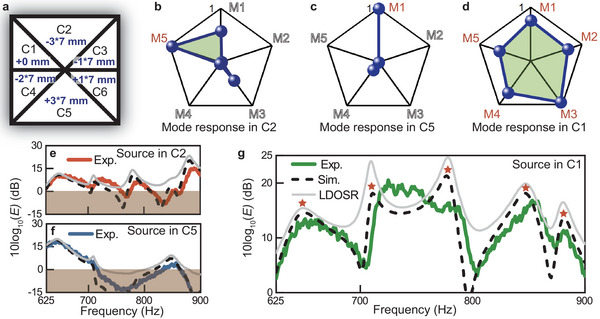
Broadband emission enhancement with all target QBICs. a) Modulation of the depths of the six cavities. b–d) The mode responses to the five QBICs for the acoustic source in cavities C2, C5, and C1, respectively. e–g) Measured (red, blue, and green lines) and simulated (black dashed lines) results of the Purcell factors (*E*) with the acoustic source in cavities C2, C5, and C1, respectively. The grey lines represent the corresponding LDOSR.

As demonstrated in **Figure** **5**a, a significant depth difference is introduced between cavities C2 and C5 (modulation units of –3 and +3), preventing them from simultaneously exhibiting strong mode responses to QBIC1 and QBIC5. Meanwhile, the depths of the remaining four cavities (C1, C3, C4, and C6) are designed to have relatively small depth differences from each other (modulation units of 0, –1, –2, and +1), but substantial differences from cavities C2 and C5. This design results in weakened mode responses to QBIC3 for both cavities C2 and C5. Consequently, together with the weak mode responses to QBIC2 and QBIC4 due to the original mode characteristics, the overall mode responses of cavities C2 and C5 to the five QBICs are weakened Figure 5b‐c, while the overall mode responses of the remaining four cavities are enhanced. During the modulations, the modulation unit of –2 for cavity C4 is designed, which ensures that the vertical symmetry properties of cavities C1 and C3 are superior to those of cavities C4 and C6, allowing for the relative dominance of cavities C1 and C3 in constricting QBIC2 and QBIC4. Thus, cavities C1 and C3 achieve relatively strong mode responses to the five QBICs, exhibiting 5 and 4 highly responsive QBICs for cavities C1 Figure 5d and C3 (see Figure S15, Supporting Information), respectively. In contrast, cavities C2 and C5 both exhibit only 1 highly responsive QBIC. Additionally, cavities C4 and C6 both possess 2 highly responsive QBICs. These results validate the effectiveness of the presented modulation strategy. The detailed mode responses and emission properties referring to cavities C3, C4, and C6 are demonstrated in Figure S15 (Supporting Information). The eigenvalues and pressure fields of the five QBICs are illustrated in Figure S16 (Supporting Information). Furthermore, the unit depth difference of 7 mm Figure 5a is designed to achieve possibly wider overall operating bandwidth of the collective QBICs (see more discussions in Section N, Supporting Information).

Figure 5e,f illustrates the emission performances for an acoustic source positioned at the bottom centers of cavities C2 and C5, respectively. Low emission enhancement or restricted emission are observed near the frequencies corresponding to the QBICs with weakened mode responses, resulting in inferior overall emission performances in broadband. In Figure 5e, the maximum experimental results of 10log_10_(*E*) is 14.8 dB at 887 Hz, and restricted emission occurs within 759–778 Hz and 815–866 Hz. In Figure 5f, the maximum experimental results of 10log_10_(*E*) is 17.7 dB at 644 Hz, and restricted emission occurs within 716–833 and 863–900 Hz. In comparison, when the acoustic source is placed in cavity C1, the Purcell effect for broadband emission enhancement is achieved owing to its strong mode responses to all five QBICs. Experimental results demonstrate that the Purcell factors are consistently exceeding 1 within the frequency range from 625 to 900 Hz, and the maximum value of 10log_10_(*E*) achieves 20.3 dB at 725 Hz Figure 5g. The experimental and simulation results of the Purcell factors agree well with each other. The calculated LDOSR provides the emission potential without intrinsic loss. In addition, we further verify that the broadband Purcell effect demonstrated here is overwhelmingly contributed by the QBICs, rather than the normal resonances of the presented system (see Figure S18, Supporting Information). These findings validate the capability of collective QBICs with comprehensively controlled mode responses in inducing the broadband Purcell effect.

Here, we should also acknowledge the potential limitation of our approach when further pursuing wider operating bandwidths. The inherent relationship between intrinsic loss and the narrowness of resonant structures is a primary factor limiting the Purcell factors, the number of QBICs, and the achievable bandwidth. The maximum operating bandwidth achievable by our proposed method of utilizing collective QBICs for broadband Purcell effect is proportional to the number of QBICs. For instance, ten QBICs with similar characteristics to the QBICs in Figure 5 can extend the operating bandwidth to around one octave. However, the suitable number of QBICs is primarily limited by the system's intrinsic loss. In the scenario of ten QBICs, more component cavities are required, and this will lead to narrower cavity dimensions, thereby resulting in increased intrinsic loss^[^ ^57^ ^]^ (see Figure S19, Supporting Information) and reduced Purcell factors. It is suggested that appropriately increasing the size of the QBIC‐supporting system can mitigate this limitation.

## 3 Conclusion

In summary, our study demonstrates the strong and broadband Purcell effect induced by collective QBICs. The low radiative decay rates of QBICs combined with their strong mode responses at the acoustic source provide exceptional potential for enhancing the local density of states and facilitating the strong Purcell effect. As a proof of concept, we propose a six‐cavity coupled system capable of supporting five collective (Q)BICs. The resonance‐interaction mechanisms underlying the formation of these five QBICs are systematically analyzed, revealing their fundamental symmetry‐related characteristics. By employing a symmetry‐modulation strategy, we enable an acoustic source within the six‐cavity coupled system to exhibit strong mode responses to all five QBICs, thereby achieving the Purcell effect for broadband emission enhancement. This work explores the modulation of collective mode responses and the broadband Purcell effect induced by collective QBICs, laying a solid foundation for advanced broadband emission control. These findings have profound implications for wave physics, particularly deepening the understanding of collective acoustic BICs^[^ ^8^, ^27^, ^29^ ^]^ and the broadband Purcell effect.^[^ ^3^, ^46^, ^66^ ^]^

## 4 Experimental Section

### Device fabrication and measurement

The experimental samples were fabricated by 3D‐printing technology with a manufacturing precision of 0.1 mm and were connected with the waveguide's opening Figure 4b. A melamine‐foam acoustic wedge with a length of 960 mm was placed at the waveguide's end to create an anechoic boundary condition. When measuring the resonant system, a loudspeaker (acoustic source) was placed at the bottom center of a certain cavity and emitted white noise. When measuring an empty tube, the loudspeaker was mounted at the bottom center of a square‐sectional tube with a side length matching that of the waveguide. With this setup, the amplitudes of the emitted sound waves can be measured by a 1/4‐inch condenser microphone (Brüel & Kjær Type 4187) situated at position 350 mm in front of the tested structure.

The reflection coefficients were measured using the impedance tube method complying with ASTM C384‐04(2011) and ASTM E1050‐12. The experimental equipment includes a square impedance tube with a side length of 10.2 cm, two 1/4‐inch condenser microphones (Brüel & Kjær Type 4187) situated at designated positions to obtain the amplitude and phase of the pressure distribution. A digital signal (white noise) generated by the computer was sent to the power amplifier (Brüel & Kjær Type 2734) and then powered the loudspeaker. By analyzing the signals from the two microphones, the reflection coefficients can be obtained.

### Numerical Simulation

The numerical calculations of the eigenfields and the Purcell factors were performed using the commercial finite element software COMSOL Multiphysics with the preset “Pressure Acoustics, Frequency Domain” module. The domain material is air, with the following properties: static air density, *ρ* = 1.21 kg · m^−3^; sound speed, *c* = 343 m · s^−1^; and dynamic viscosity, *μ* = 1.81 × 10^−5^ N · S · m^−^^2^. The preset environmental temperature is T = 293.15K (20 °C). Due to the significant impedance contrast between the sample photosensitive resin and air, the boundaries of the sample (with a thickness of 5 mm) can be considered acoustically rigid. Consequently, in the simulations, the structural boundaries are set as “Sound Hard Boundary.” Additionally, the end of the radiation channel is set as “Perfectly Matched Layer.” The intrinsic loss is calculated by introducing the “Thermoviscous Boundary Layer Impedance,” where the mechanical condition of the structure's boundaries is set as “No slip,” and the thermal condition is set as “Isothermal.”

In calculating the eigenfrequencies, the qualities of radiation loss (referred to *γ*_0_) are focused, without considering intrinsic (thermal‐viscous) loss. Thus, by conducting an “Eigenfrequency study,” the eigenfrequencies of the target modes can be obtained as (*ω*_0_ + *i* · *γ*_0_)/(2π), such as the eigenfrequencies shown in Figures 1, 3, and 4.

In calculating the Purcell factors, by conducting a “Frequency Domain” study, we separately simulate the emission properties of an identical sound source within an empty tube and the presented six‐cavity coupled systems. All the inner boundaries of the empty tube and the six‐cavity coupled systems are set as “Thermoviscous Boundary Layer Impedance.” The sound source employs a “Monopole Point Source” with the type of “Flow,” where the “Volume Flow Rate Out From Source” is set to 2.43 × 10^−5^ m^3^s^−1^, and the “Phase” is set to 0. With this setup, the acoustic pressure amplitude of the emitted waves for the empty tube is 1 Pa.

## Conflict of Interest

The authors declare no conflict of interest.

## Author Contributions

D.P.T., Y.L., and J.Z. conceived the idea. S.H. and T.L. carried out the theoretical analysis. S.X., T.H., and S.H. performed the measurement and the data processing. D.P.T., Y.L., and J.Z. supervised the research. S.H., T.L., and Y.L. wrote the paper with contributions from all authors.

## Supporting information

Supporting Information

## Data Availability

The data that support the findings of this study are available from the corresponding author upon reasonable request.
