# Supplementary material for: Broadband Acoustic Purcell Effect from Collective Bound States in the Continuum
Source: Adv Sci (Weinh). 2025 Feb 21;12(15):2414627. doi: 10.1002/advs.202414627 (PMC12005753; doi:10.1002/advs.202414627)
Supplement: Supplementary file 1 — Supporting Information [file ADVS-12-2414627-s001.pdf]

ADVANCED SCIENCE
Open Access

# Supporting Information

for *Adv. Sci.*, DOI 10.1002/advs.202414627

Broadband Acoustic Purcell Effect from Collective Bound States in the Continuum

*Sibo Huang, Shuhuan Xie, Tuo Liu, Tong Hao, Din Ping Tsai*, Yong Li* and Jie Zhu**

# Supplementary Material for "Broadband Acoustic Purcell Effect from Collective Bound States in the Continuum"

Sibo Huang,[1] Shuhuan Xie,[2] Tuo Liu,[3] Tong Hao,[4]
Din Ping Tsai,[1,†] Yong Li,[2,‡] and Jie Zhu,[2,§]

[1]*Department of Electrical Engineering, City University of Hong Kong, Hong Kong SAR, China*

[2]*Institute of Acoustics, Tongji University, Shanghai 200092, China*

[3]*Key Laboratory of Noise and Vibration Research, Institute of Acoustics, Chinese Academy of Sciences, Beijing 100190, China*

[4]*College of Surveying and Geo-Informatics, Tongji University, Shanghai 200092, China*

†dptsai@cityu.edu.hk ‡yongli@tongji.edu.cn §jiezhu@tongji.edu.cn

**The PDF file includes:**

Supplementary Text

Figure S1 to S19

## Section A

**Reflection spectra of the structures.** We have demonstrated the realization of five QBICs by breaking the symmetry properties of the presented system in Figure 1-5. Since BICs are completely isolated modes, they will vanish in the reflection spectra of the BIC-supporting systems. Nevertheless, when modulating the BIC-supporting systems to achieve QBICs, reflection dips will appear in the reflection spectra at the QBICs. The intrinsic losses in the presented system are relatively small, and thus the QBICs appear in the simulation and experimental reflection spectra at the frequencies approximating their real parts of eigenvalues. Figure S1a-b illustrates the experimental sample and the reflection spectrum of the structures shown in Figure 1. Only QBIC3 ($\sim$775 Hz) is observed in the reflection spectrum with a small reflection dip, while BIC1 ($\sim$689 Hz), BIC2 ($\sim$773 Hz), BIC4 ($\sim$780 Hz), and BIC5 ($\sim$794 Hz) vanish. Figure S1c-d corresponds to the structure shown in Figure 3, where the depths of cavities C4, C5, and C6 are increased to break the transverse symmetries. Thus, BIC1 and BIC5 turn into QBIC1 ($\sim$634 Hz) and QBIC5 ($\sim$783 Hz), leading to two additional narrow reflection dips [Figure S1d]. The preserved vertical symmetries of the structure allow BIC2 ($\sim$635 Hz) and BIC4 ($\sim$756 Hz) to maintain pure BICs, so BIC2 and BIC4 are vanishing in the reflection spectrum. Figure S1e-f corresponds to the structure shown in Figure 4, where we further break the vertical symmetries of the structure and obtain five QBICs. In this scenario, five reflection dips can be observed at the five QBICs. Figure S1g-h corresponds to the structure shown in Figure 5. Furthermore, we conducted simulations to comprehensively analyze all the modes near the frequencies of (Q)BICs, confirming that the $Q_{\text{rad}}$ of other nearby modes are at least more than an order of magnitude lower than those of the (Q)BICs. Consequently, the bandwidths of reflection dips for these nearby modes will be significantly wider than those observed near the QBICs in Figure S1. This analysis further validates that the relatively narrow reflection dips near QBICs shown in Figure S1 are associated with the QBICs.

Based on the reflection spectra, we can experimentally calculate the quality factors ($Q_{\text{exp}}$) of the QBICs by identifying their full width at half-maximum (FWHM). The structure in Figure 1 has $Q_{\text{exp}} = 103$ (QBIC3); The structure in Figure 3 has $Q_{\text{exp}} = 61$ (QBIC1), 63 (QBIC3), 79 (QBIC5); The structure in Figure 4 has $Q_{\text{exp}} = 60$ (QBIC3), 69 (QBIC4), 51 (QBIC5); The structure in Figure 5 has $Q_{\text{exp}} = 21$ (QBIC1), 59 (QBIC2), 52 (QBIC3). The

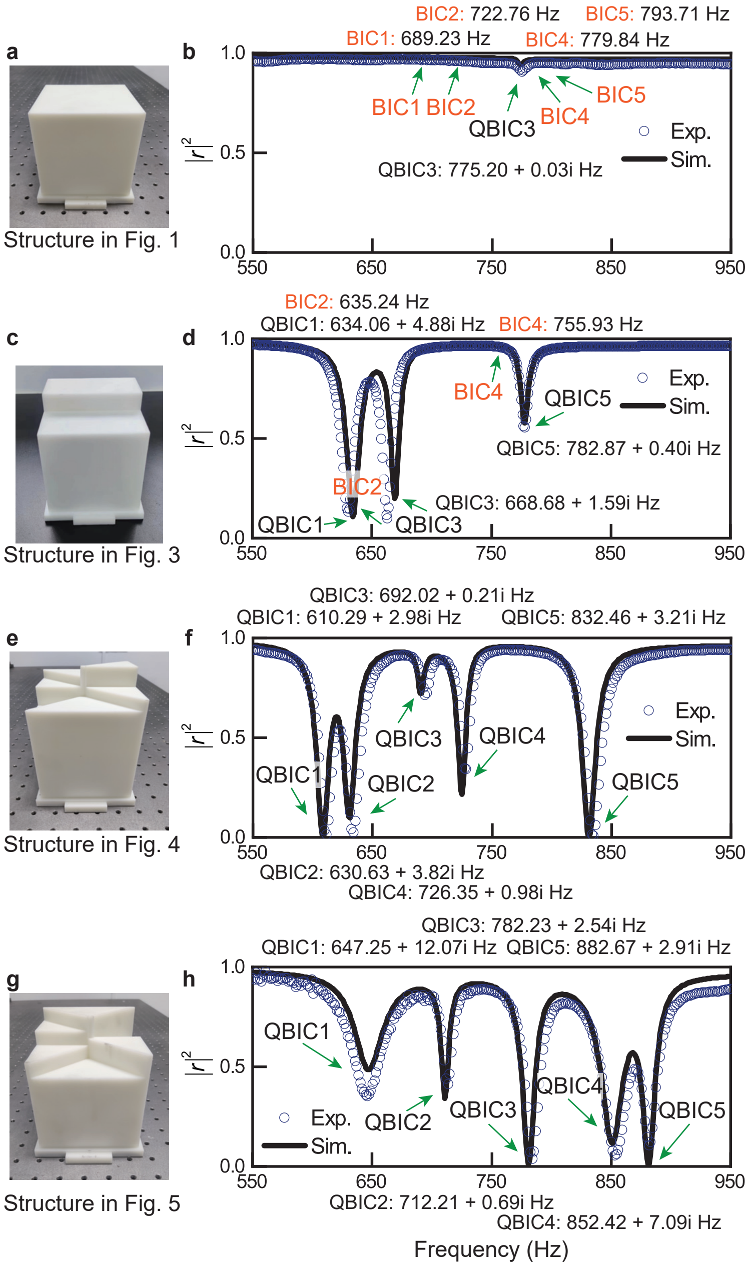


FIG. S1. Reflection spectra of the QBIC-supporting systems. a-b) Sample and reflection spectrum of the structure in Figure 1. c-d) Sample and reflection spectrum of the structure in Figure 3. e-f) Sample and reflection spectrum of the structure in Figure 4. g-h) Sample and reflection spectrum of the structure in Figure 5. $|r|$ represents the amplitudes of reflection coefficients.

suitable values of quality factors should contribute to a strong Purcell effect, and meanwhile, allow the working bandwidths of the QBICs to span the inter-mode frequency intervals. Here, the quality factors of the pure BICs cannot be obtained experimentally from the

reflection spectra since the pure BICs cannot be excited and tested from the far field. In addition, when the modes are not well separated and identified in the frequency domain (i.e., the FWHM of each mode is larger than the inter-mode frequency difference), the calculation of experimental quality factors of the modes is not available. When ignoring the intrinsic loss, the radiation quality factors ($Q_{\mathrm{rad}}$) of the QBICs can be calculated based on their eigenvalues ($Q_{\mathrm{rad}} = \frac{\omega_0}{2\gamma_0}$). Specifically, the structure in Figure 1 has $Q_{\mathrm{rad}} = 12920$ (QBIC3); The structure in Figure 3 has $Q_{\mathrm{rad}} = 65$ (QBIC1), 210 (QBIC3), 978 (QBIC5); The structure in Figure 4 has $Q_{\mathrm{rad}} = 102$ (QBIC1), 82.5 (QBIC2), 1647.7 (QBIC3), 370.6 (QBIC4), 129.7 (QBIC5); The structure in Figure 5 has $Q_{\mathrm{rad}} = 26.8$ (QBIC1), 516.1 (QBIC2), 154 (QBIC3), 60.1 (QBIC4), 151.7 (QBIC5).

## Section B

**Experimental verification of the presence of BICs based on vanishing linewidth phenomena.** Since BICs are completely isolated modes that disappear in reflection spectra, to experimentally indicate the presence of BICs and QBICs, one commonly used method is observing the zero (vanishing) linewidth phenomenon in the evolution process from QBICs to BICs in reflection spectra. Figure S2 demonstrates the numerically calculated reflection diagram illustrating the vanishing linewidth phenomena. We experimentally verified this result, as shown in Figure S3. Focusing on QBIC1 and QBIC5 illustrated in Figure 3, they exhibit reflection dips with certain reflection bandwidths (referred to the results in Figure S1e). By changing the unit depth difference from 20 mm to 0 mm, we can trace the evolution process of the reflection dips corresponding to QBIC1 and QBIC5 from pure BIC1 and BIC5, where the presence of BIC1 and BIC5 are characterized by the vanishing linewidth phenomena.

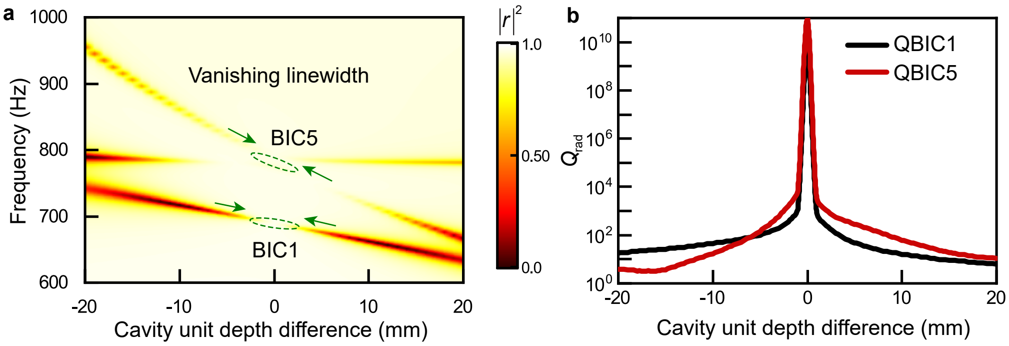


FIG. S2. Characterization of the BICs. a) BICs and QBICs characterized by the vanishing linewidth phenomena. The BIC-supporting system follows the cavity depth modulation pattern of the structure in Figure 3a but with various unit depth differences. b) The $Q_{\mathrm{rad}}$ of QBIC1 and QBIC5 with varying cavity unit depth differences.

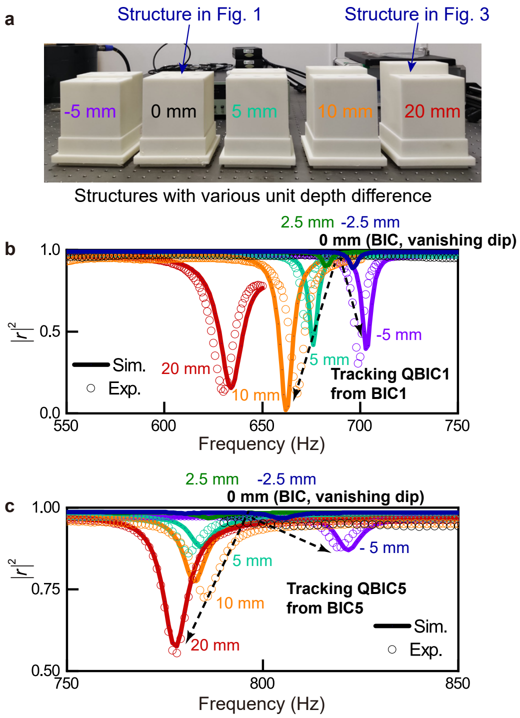


FIG. S3. Tracing QBICs from BICs. a) BIC-supporting systems with various unit depth differences. The depths of cavities C4-C6 are larger than cavities C1-C3 with a designed unit depth difference. b) Tracing QBIC1 from BIC1. c) Tracing QBIC5 from BIC5.

**Theoretical verification of the presence of BICs based on eigenvalue evolution analysis.** Figure S4 demonstrates that the five modes are all evolved from pure BICs whose imaginary parts achieve zero when the system's unit depth difference is close to zero, and thus these five modes are QBICs when they deviate from the pure BICs. For the presented structure shown in Figure 5a and Figure S1h, its unit depth difference is 7 mm. Accordingly, we identify that the five eigenvalues of the modes demonstrated in Figure S4 are equal to those demonstrated in Figure 5a, confirming those relevant modes are QBICs.

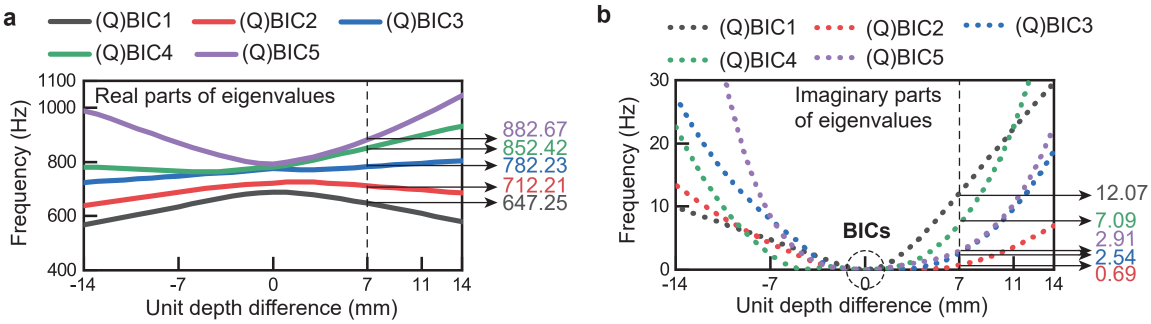


FIG. S4. Verification of QBICs in Figure 5 through eigenvalue evolution analysis. a-b) Evolution of the real and imaginary parts of the eigenvalues along with varying unit depth differences. The BIC-supporting system follows the cavity depth modulation pattern of the structure in Figure 5.

## Section C

**Schematic illustration of the implementation of the broadband Purcell effect induced by collective QBICs.** As shown in Figure S5, an acoustic source within a multi-resonator system that supporting collective BICs. When a single resonator is tuned to exhibit strong mode responses to all target QBICs at different frequencies, the acoustic source within the single cavity can achieve broadband emission enhancement.

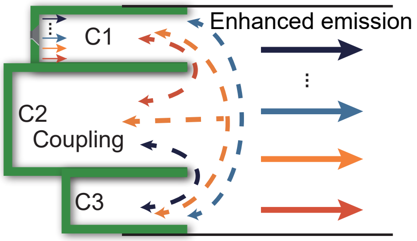


FIG. S5. Conceptual implementation demonstration of the enhanced emission at QBICs.

## Section D

**Irreducible classification of the five (Q)BICs based on eigenfield distributions.** The eigenfield distributions of five (Q)BICs can be classified into the irreducible representations of the $C_{2v}$ group. Table S1 demonstrates the character table for the $C_{2v}$ point group, which consists of four symmetry operations as follows. The identity operation $E$: This operation leaves the structure unchanged. The $C_2$ rotation: This operation corresponds to a rotation of the structure by $\pi$ radian. The mirror reflection $\sigma_x$: This operation reflects the structure with respect to the x-axis. The mirror reflection $\sigma_y$: This operation reflects the structure with respect to the $y$-axis.

By applying the four symmetry operations mentioned above to the eigenfield distributions of the five (Q)BICs [Figure S6] and comparing the results with Table S1, we can determine that BIC1 corresponds to the $B_2$ irreducible representation of the $C_{2v}$ group; BIC2 corresponds to the $B_1$ irreducible representation of the $C_{2v}$ group; QBIC3 corresponds to the $A_1$ irreducible representation of the $C_{2v}$ group; BIC4 corresponds to the $A_2$ irreducible representation of the $C_{2v}$ group; BIC5 corresponds to the $B_2$ irreducible representation of the $C_{2v}$ group.

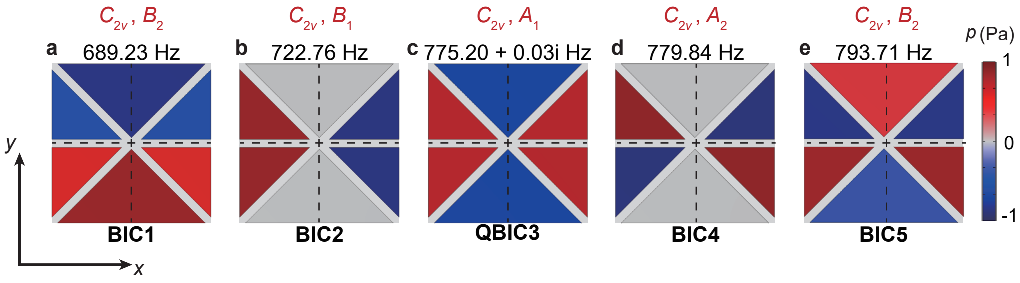


FIG. S6. The irreducible representations of the five (Q)BICs. The black dashed lines represent mode-distribution symmetric lines of eigenfields for the (Q)BICs.

TABLE S1. The character table for the $C_{2v}$ point group.

| $C_{2v}$ | $E$ | $C_2$ | $\sigma_x$ | $\sigma_y$ |
|---|---|---|---|---|
| $A_1$ | 1 | 1 | 1 | 1 |
| $A_2$ | 1 | 1 | -1 | -1 |
| $B_1$ | 1 | -1 | 1 | -1 |
| $B_2$ | 1 | -1 | -1 | 1 |

## Section E

**Three-dimensional illustration of the pressure fields shown in Figure 1d-h.** Figure S7 demonstrates that the BICs have no far-field radiation to the outside of the presented six-cavity coupled system. Besides, the bottoms of the cavities are acoustically hard walls, generally supporting the amplitude (the maximum value) of acoustic pressure. Consequently, inside each cavity of the presented system, the pressure fields at the bottom of the cavity are relatively intensive. The structures in Fig 3, Figure 4, and Figure 5 also have similar pressure-distribution features. Therefore, we set the acoustic source at the bottom centers of the cavities for the pursuit of stronger emission enhancement.

**Amplitudes of the pressure fields shown in Figure 1-4.** Figure S8-S10 demonstrate the amplitudes of the pressure fields of the five (Q)BICs in Figure 1-4 from the view of the structure's bottom. Since the antinodes of the pressure fields are located at the bottoms of the cavities, the distributions of pressure amplitudes [Figure S8-S10] and pressure real parts [Figure 1-4] have consistent trends at the bottoms of the cavities.

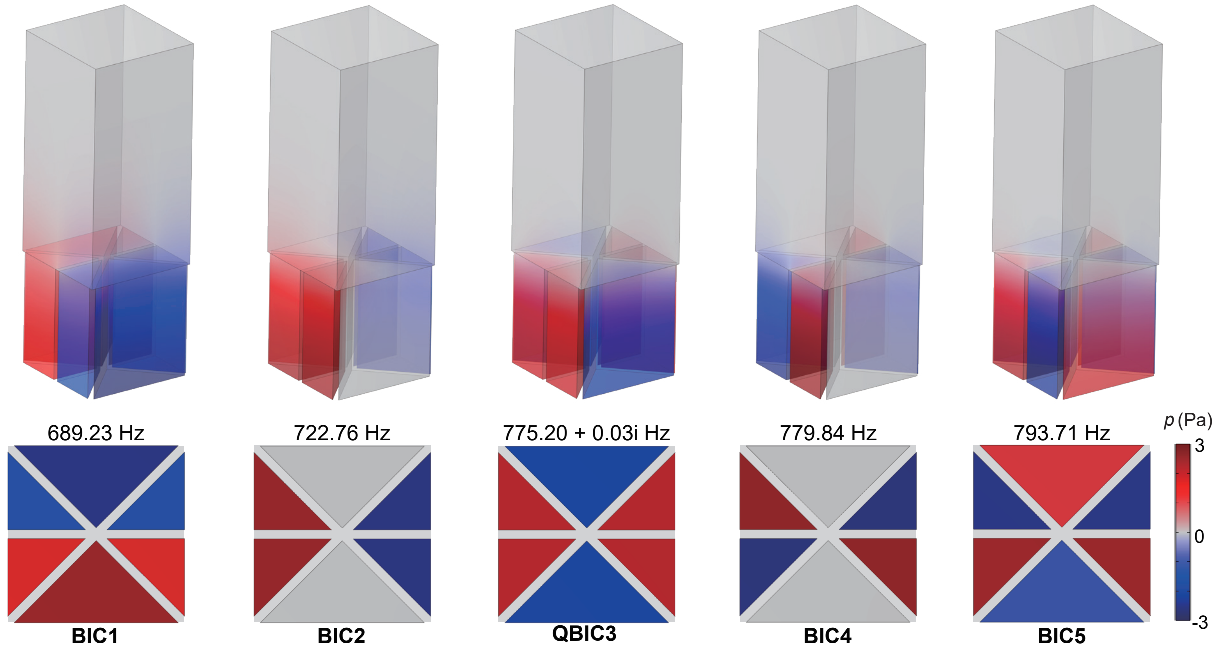


FIG. S7. Pressure fields and eigenfrequencies of the five (Q)BICs supported by the six-cavity coupled system presented in Figure 1. The upper panel illustrates the three-dimensional pressure fields, and the corresponding pressure fields from the view of the structure's bottom are shown below. The top boundaries of the radiation channels are non-reflective.

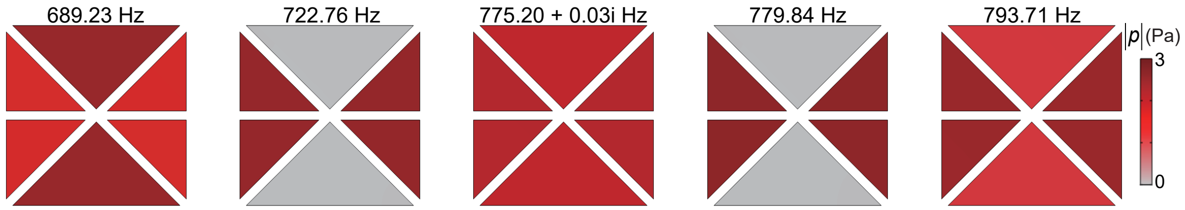


FIG. S8. Pressure fields' amplitudes of the five (Q)BICs in Figure 1 (structure's bottom view) and their eigenfrequencies.

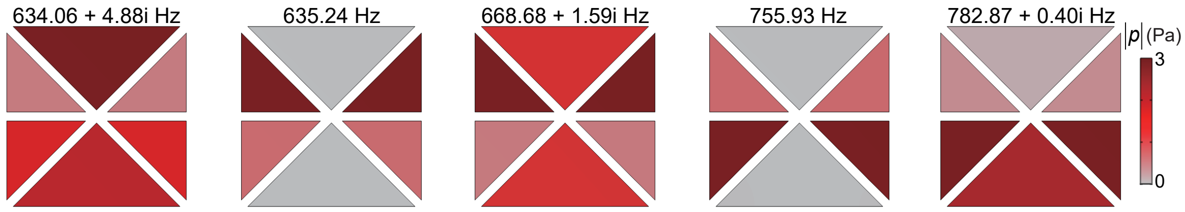


FIG. S9. Pressure fields' amplitudes of the five (Q)BICs in Figure 3 (structure's bottom view) and their eigenfrequencies.

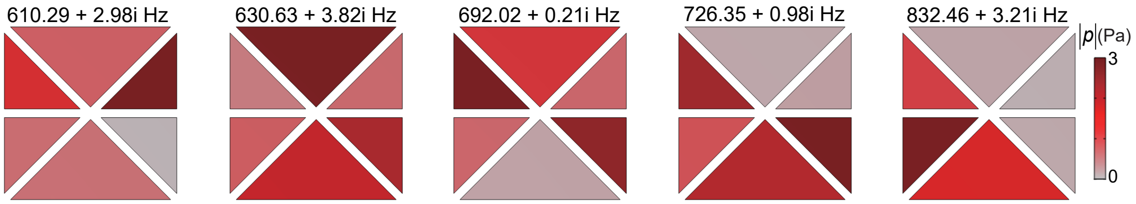


FIG. S10. Pressure fields' amplitudes of the five QBICs in Figure 4 (structure's bottom view) and their eigenfrequencies.

## Section F

**Coupling types and eigenfrequency analysis of the presented six-cavity coupled system.** Figure S11 illustrates the six coupling types for the six-cavity coupled system presented in Figure 1b.

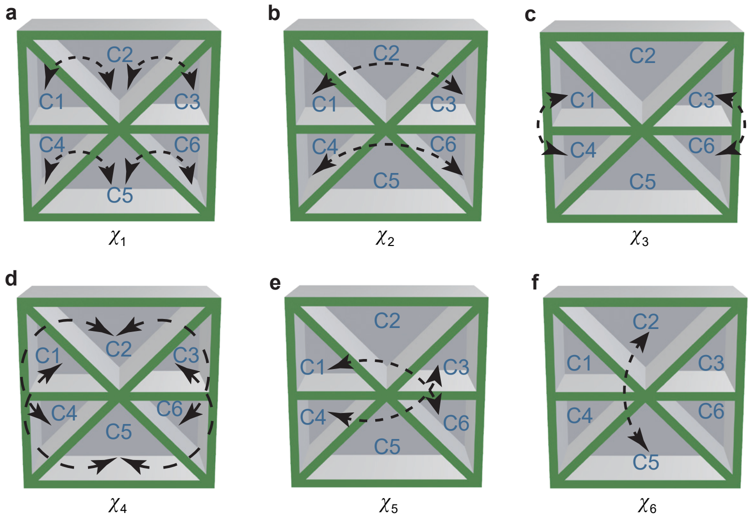


FIG. S11. Coupling types for the six-cavity coupled system presented in Figure 1b. a-d) The six types of couplings ($\chi_1$ to $\chi_6$).

**Analysis of the near-field and radiation couplings.** The near-field coupling factors ($\kappa$) and radiation coupling factors ($\gamma_{\mathrm{fc}}$) can be calculated based on the eigenvalues of resonant systems. The values of $\kappa$, whether positive or negative, characterize the nature of the modes formed by in-phase and out-of-phase couplings [64]. Specifically, a negative value of $\kappa$ indicates that the in-phase coupled mode has energy lower than that of the out-of-phase mode, whereas a positive $\kappa$ suggests the opposite. For the structure presented in Figure 1, the eigenvalues $((\omega_0 + i \cdot \gamma_0)/(2\pi))$ of individual cavity C1 and individual cavity C2 are

$(762 + 45i)$ Hz and $(746.6 + 98.2i)$ Hz, respectively. Cavities C3, C4, and C6 have the same eigenvalue as cavity C1; cavity C5 has the same eigenvalue as cavity C2. Thus, we obtain $\gamma_1 = \gamma_3 = \gamma_4 = \gamma_6 = 45 \times 2\pi$ Hz, $\gamma_2 = \gamma_5 = 98.2 \times 2\pi$ Hz. Accordingly, the radiation coupling factors from $\gamma_{\text{fc1}}$ to $\gamma_{\text{fc6}}$ can be calculated, where $i\gamma_{\text{fc1}} = i\gamma_{\text{fc4}} = i\sqrt{\gamma_1\gamma_2} = 66.5i \times 2\pi$ Hz, $i\gamma_{\text{fc2}} = i\gamma_{\text{fc3}} = i\gamma_{\text{fc5}} = i\gamma_1 = 45i \times 2\pi$ Hz, $i\gamma_{\text{fc6}} = i\gamma_2 = 98.2i \times 2\pi$ Hz.

Furthermore, based on the expressions of eigenvalues derived in Table I, the six unknowns corresponding to the six near-field coupling factors are determined by solving this system of six equations. In Figure 1, we have demonstrated the eigenvalues of the five (Q)BICs, and the sixth eigenvalue is $(818 + 399.3i)$ Hz. The correspondence between the six eigenvalue expressions in Table I and the six calculated eigenvalues can be theoretically established through the eigenfields. For instance, the eigenvectors of $\sigma_1$ and $\sigma_2$ are {-1, 0, 1, -1, 0, 1} and {1, 0, -1, -1, 0, 1}, respectively. The first eigenvector corresponds to the eigenfield of BIC2, where there is no contribution from cavity C2 and C5, and the eigenfield phases between cavity C1 (-1) and cavity C4 (-1) should be identical. In addition, the second eigenvector corresponds to BIC4, where the eigenfield phases between cavity C1 (1) and cavity C4 (-1) should be opposite. However, the calculation of the eigenvectors for $\sigma_3$-$\sigma_6$ poses significant mathematical challenges. In Figure 2, we have validated that $\sigma_5$ corresponds to QBIC3 and $\sigma_6$ corresponds to the lossy mode through numerical analysis. Subsequently, by comparing the magnitudes of $\sigma_3$ and $\sigma_4$, we can identify that they correspond to BIC1 and BIC5, respectively. After obtaining the correspondences of the six eigenvalue expressions and eigenvalue results, we can theoretically calculate the six near-field coupling factors. Since the six equations constitute a rather complex nonlinear system, we utilize the "fmincon" algorithm in MATLAB to numerically solve the near-field coupling factors. With the purely real eigenvalues calculated from simulations and the Hamiltonian matrix, we comprehensively prove the existence of the BICs and their underlying formation mechanisms.

## Section G

**Visual transition from the main figures to the subfigures in Figure 2.** The main figures of Figure 2b and Figure 2d may appear relatively flat and exhibit a wider bandwidth compared to those observed in the corresponding subfigures. However, this perceived discrepancy stems solely from the visual effects caused by the magnification differences between the overall and localized views. Figure S12 demonstrates the gradual visual transition from the main figures to the subfigures.

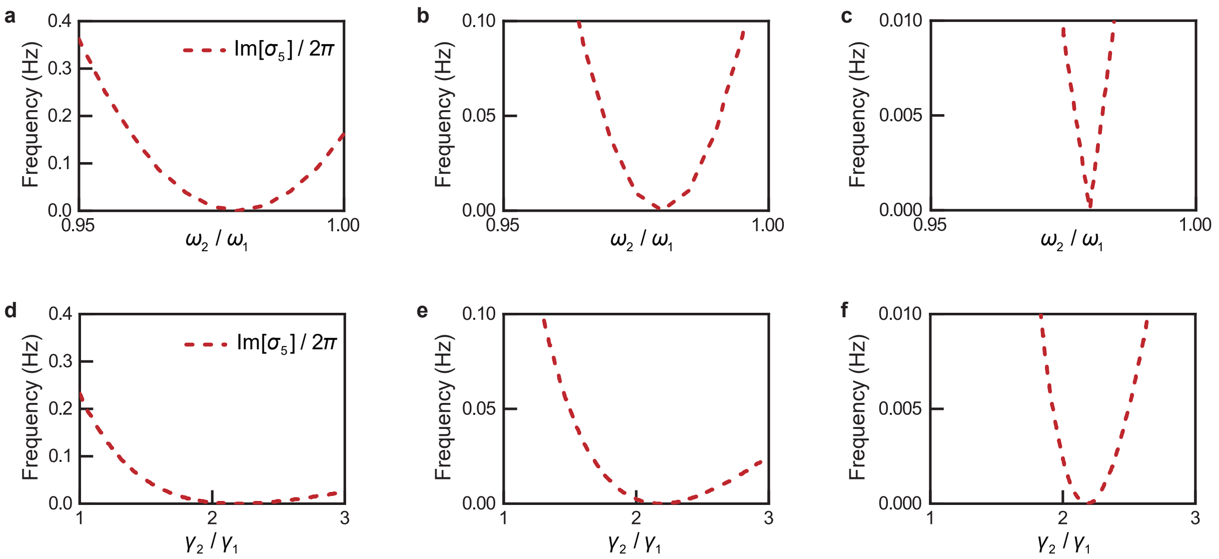


FIG. S12. Auxiliary figures demonstrating the transition from the main figures to the subfigures in Figure 2. a-c) The imaginary parts of $\sigma_5$ with varying ratios between $\omega_2$ and $\omega_1$. All the illustrated curves in a-c) are identical but only with different ranges displayed on the vertical axis. d-f) The imaginary parts of $\sigma_5$ with varying ratios between $\gamma_2$ and $\gamma_1$. All the illustrated curves in d-f) are identical but only with different ranges displayed on the vertical axis.

## Section H

**Influence of different types and sizes of acoustic sources on the Purcell effect.** Membrane-vibration-based loudspeakers, such as dynamic, electrostatic, and balanced-armature types, exhibit nearly identical acoustic source strength $A_\mathrm{s} = S_0 u_\mathrm{a}$ within different surrounding structures (such as within an empty tube or a multi-resonator structure) under the same driving voltage, where $u_\mathrm{a}$ is the amplitude of the membrane's surface vibration

velocity, $S_0 = 4\pi R_0^2$ is the effective surface vibration area of the loudspeaker, and $R_0$ is the loudspeaker's effective radius. This result is attributed to their electroacoustic impedance characteristics and electromechanical coupling mechanisms. For these loudspeakers, the radiation impedance is several orders of magnitude smaller than the sum of electrical impedance and mechanical impedance. While the surrounding structures of a loudspeaker can alter its radiation impedance, the overall electroacoustic impedance remains almost unchanged, resulting in a nearly constant $A_{\mathrm{s}}$. Therefore, as long as these loudspeakers exhibit nearly identical $A_{\mathrm{s}}$ within different structures, the Purcell effect remains fundamentally similar.

However, special types of loudspeakers, such as air-compression loudspeakers, have distinct electromechanical coupling mechanisms and electroacoustic impedance characteristics, which can lead to variations in the Purcell effect.

In our experiments, we employed a commonly seen dynamic loudspeaker, which presents almost identical acoustic source strength within the empty tube and QBIC-supporting structures under the same driving voltage. Accordingly, in our simulations, we also set a "Monopole Point Source" with a constant $A_{\mathrm{s}}$. The experimental and simulation results demonstrated good agreement.

In conclusion, for most membrane-vibration-based loudspeakers, their sizes and types do not significantly affect the performance of the Purcell effect. However, some special loudspeakers, such as air-compression loudspeakers, can lead to variations in the performance of the Purcell effect.

## Section I

**Measured sound pressure with the structures and the empty tube.** Figure S13 demonstrates the broadband emission enhancement with measured sound pressure data for the system conditions shown in Figure 4c and Figure 5g. Compared to an empty tube, the presented structures achieve significantly enhanced emission sound pressure of the acoustic source within certain frequency bands [Figure S13a-b]. By achieving strong mode responses of more QBICs, the bandwidth of emission enhancement becomes broader [Figure S13c].

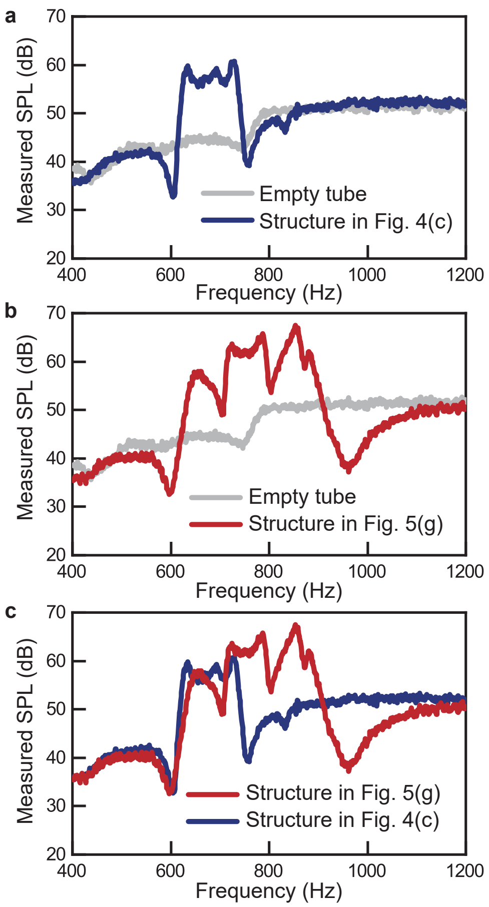


FIG. S13. Demonstration of broadband emission enhancement with measured sound pressure data. a) Measured emitted sound pressure of an acoustic source with an empty tube and the structure in Figure 4c. b) Measured emitted sound pressure of the acoustic source with the empty tube and the structure in Figure 5g. c) Comparison of the measured emitted sound pressure with structures in Figure 5g and Figure 4c. The white noise frequency range (400-1200 Hz) and the driving voltage (1.414 Vrms) were kept identical for all measurements.

## Section J

**Discussions on the scenario of multiple acoustic sources.** For the scenario involving two or more acoustic sources in different cavities of a multi-cavity system, the sources will act as preset boundary conditions. Consequently, the original modes of the multi-cavity system may be disrupted by these preset boundary conditions.

As the structure in Figure 4a, when a single acoustic source is placed in cavity C1, the pressure amplitudes of the overall eigenfield can be excited by the acoustic source propor-

tional to the mode responses, where the distribution of the system's eigenfield is maintained. However, when two acoustic sources are placed respectively in cavities C1 and C2, two preset boundary conditions are created at the bottoms of the two cavities. For two identical sources, the pressure amplitude and phase at the bottoms of cavities C1 and C2 must be the same. For two different sources, the pressure amplitudes and phases at the bottoms of cavities C1 and C2 should have a certain amplitude-phase difference. In these circumstances, this kind of dual-source boundary conditions will disrupt most of the modes of the system in Figure 4a. Therefore, a single source is more advantageous than multiple simultaneous sources in effectively exciting more QBICs for the more broadband Purcell effect. In addition, in practical applications, using a single source is a simpler and more feasible setup.

**Discussions on the scenario of separate QBICs supported by different uncoupled cavities.** In theory, employing separate QBICs supported by different uncoupled cavities to achieve broadband Purcell effects is feasible. For instance, still assuming a six-cavity system, cavity C1 and cavity C2 support BIC1, independent of the other four cavities. Similarly, cavity C3 and cavity C4 support BIC2, and cavity C5 and C6 support BIC3, with the corresponding modes independent of the other four cavities. Then, using three loudspeakers, respectively, located at Cavities C1, C3, and C5, the three QBICs can simultaneously be excited, leading to the broadband Purcell effect. However, practical implementation presents significant challenges. For instance, ensuring that cavities do not couple would require significantly increased distances between them, compromising the compactness of the structure. Moreover, to achieve relatively separate QBICs, the resonant frequencies of the component cavities should have large frequency intervals. This will lead to dips in the emission enhancement curves, preventing the achievement of broadband emission enhancement. Therefore, to maintain both compactness and continuous broadband enhancement, it is necessary to utilize collective QBICs rather than separate QBICs.

## Section K

**Mode responses of the sound source in C2-C6 for the system presented in Figure 4a.** Figure S14 demonstrates the mode responses of cavities C2-C6 for the system shown in Figure 4. It can be observed that cavities C2-C6 are unable to support significant mode responses to all of the five QBICs. Most of them can only support strong mode responses

to one or two QBICs.

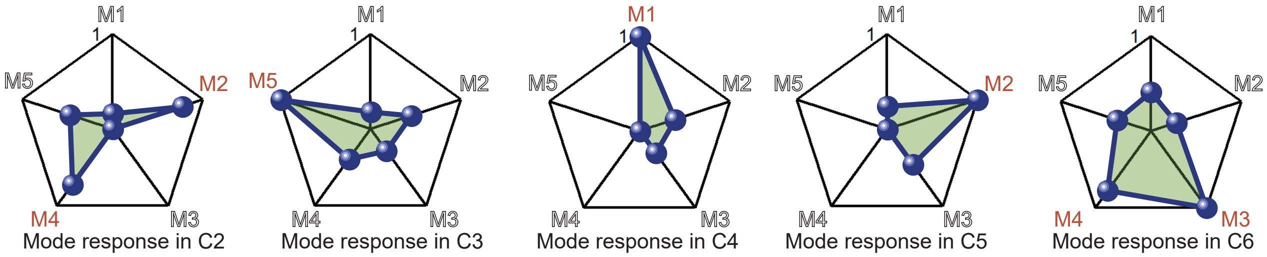


FIG. S14. The mode responses to the five QBICs for the sound source in cavity C2-C6 of the system in Figure 4.

## Section L

**Mode responses and emission properties of the sound source in C3, C4, and C6 for the system presented in Figure 5a.** Figure 5 demonstrates the weakened mode responses of cavities C2 and C5, resulting in the inferior overall emission performances when the acoustic source is placed in the two cavities. However, the reinforced mode responses of cavity C1 contribute to strong broadband emission enhancement. Here, The mode responses and the Purcell factors for the sound source in cavities C3, C4, and C6 are illustrated in Figure S15. Similarly, significant emission enhancement is achieved only at the QBICs with strong mode responses.

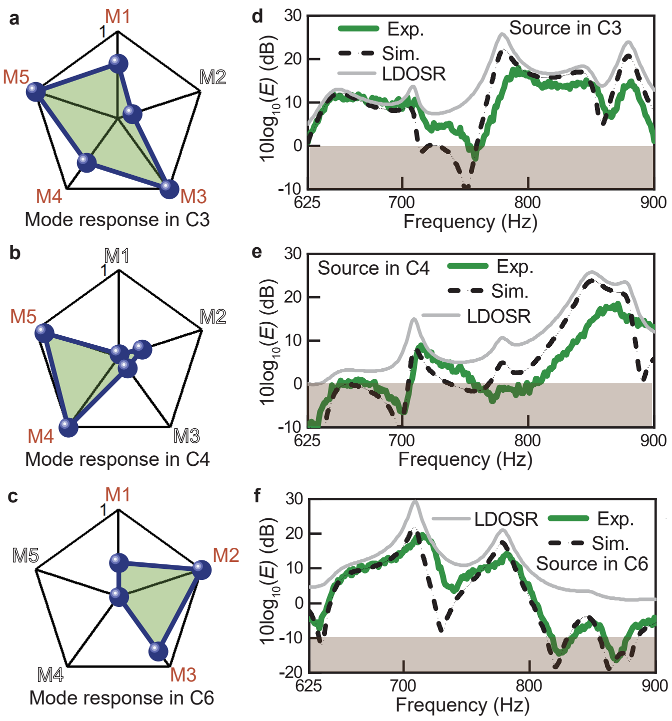


FIG. S15. Mode responses and emission properties for the system presented in Figure 5. a-c) The mode responses to the five QBICs for the sound source in cavities C3 (a), C4 (b), and C6 (c). d-f) The Purcell factors ($E$) with the sound source in cavities C3 (d), C4 (e), and C6 (f), along with the LDOSR at the sound source (grey line).

## Section M

**Eigenvalues and pressure fields of the five QBICs supported by the system in Figure 5a.** The pressure fields shown in Figure S16 demonstrate that cavity C1 (reinforced) possesses strong mode responses to all five QBICs. Besides, the pressures in cavities C2 and C5 are relatively small for most of the QBICs, showing the overall weak mode responses.

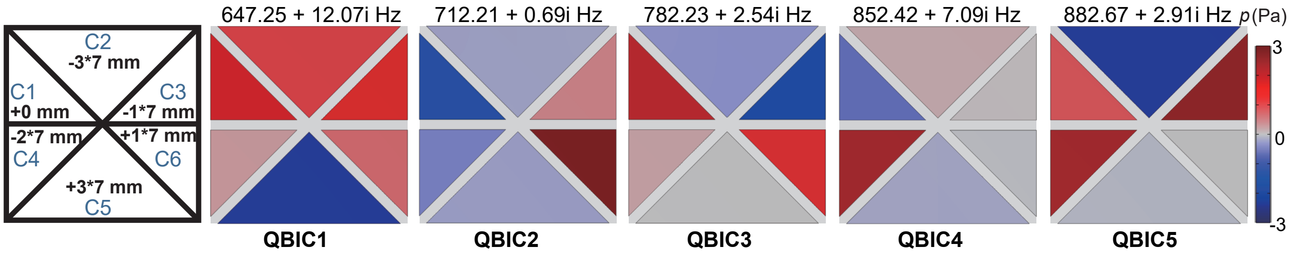


FIG. S16. Pressure fields (structure's bottom view) and eigenfrequencies of the five QBICs supported by the six-cavity coupled system in Figure 5.

## Section N

**Emission performance of the structure in Figure 5 with smaller unit depth differences.** When the nit depth difference becomes smaller, the operational bandwidth will decrease. As shown in Figure S17, the operational bandwidth of the structure in Figure 5 with a unit depth of 3 mm becomes narrower compared to the results shown in Fig. 5 with a unit depth of 7 mm. However, the Purcell factors of in Figure S17 show no significant advantages over Figure 5. Furthermore, when the unit depth difference is large, the emission restriction phenomenon occurs (shown later in Section O). As a result, the structure with a unit depth of 7 mm is a suitable design to achieve wide overall operating bandwidth as well as large Purcell factors.

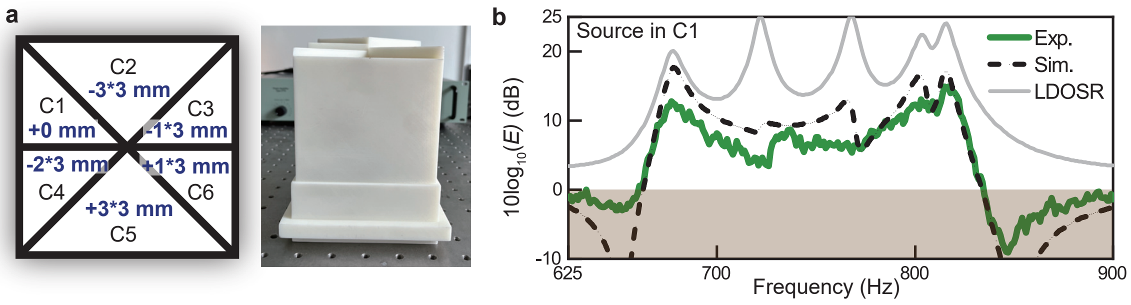


FIG. S17. Emission performance of the presented structure in Figure 5 with a unit depth of 3 mm. a) Modulation of the depths of the six cavities. The system follows the cavity depth modulation pattern of the structure in Fig. 5 but with a unit depth difference of 3 mm. b) Measured (green line) and simulated (black dashed line) results of the emission ratio ($E$) with the acoustic source in cavities C1.

## Section O

**Verification of the QBICs' contribution in the broadband Purcell effect.** To experimentally indicate that the emission enhancement performance is directly induced by the QBICs, we designed a structure with a larger unit depth difference of 11 mm [Figure S18a]. In this way, the relations between the QBICs and their induced emission-enhancement bands in the curve of Purcell factors can be identified more clearly [Figure S18b]. Figure S18c-d illustrates the evolution of five modes that can achieve BICs when the unit depth difference is around 0 mm. Therefore, it is confirmed that the five modes are (Q)BICs. Then, we identify that the five eigenvalues are equal to those demonstrated in Figure S18b, confirming those relevant modes are QBICs, instead of normal resonances. As shown in Figure S18b, it can be observed that the emission-enhancement bands are centered by the frequencies of the QBICs.

In addition, normal resonances inherently exist in the resonant systems, so we cannot exclude them in the experiments. However, we can prove that the effect of normal resonance on the broadband emission-enhancement performance is minimal. We calculated the eigenvalues of all modes within the demonstrated frequency range and found that their $Q_{\text{rad}}$ values are more than an order of magnitude lower than those of the QBICs. Besides, their frequencies are not close to the frequencies of the emission-enhancement peaks.

Based on the results above, it can be validated that these emission-enhancement bands are overwhelmingly contributed by the QBICs.

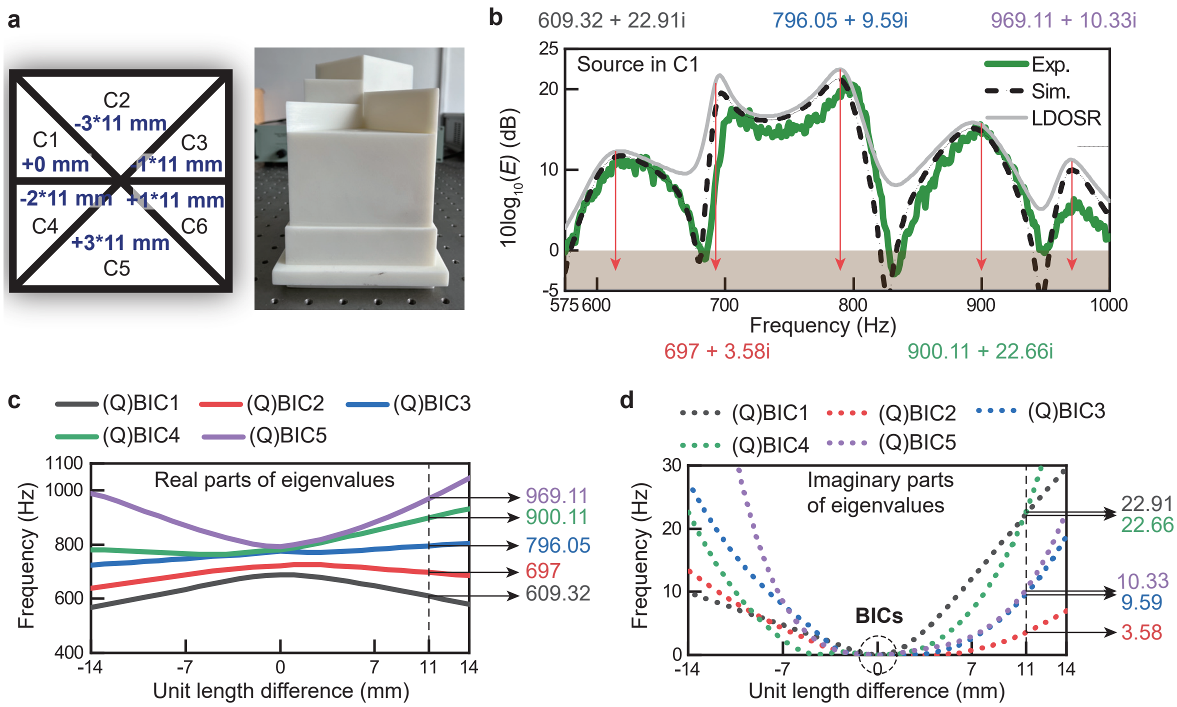


FIG. S18. Verification of QBIC-induced broadband emission enhancement. a) Modulation of the depths of the six cavities. b) Measured (green line) and simulated (black dashed line) results of the emission ratio ($E$) with the acoustic source in cavities C1. c-d) Evolution of the real and the imaginary parts of the eigenvalues along with varying unit depth differences. The BIC-supporting system follows the cavity depth modulation pattern of the structure in Figure 5a.

## Section P

**The relationship between intrinsic losses and structural narrowness of acoustic cavities.** The quantitative relationship between the cavity's cross-section parameters (narrowness) and the intrinsic loss can be calculated with the narrow region acoustics theory [57]. Considering the propagation of acoustic waves in a rectangular cavity along the x-direction as $p_0 \exp(-ik_{\mathrm{rc}}x)\exp(i\omega t)$, where $p_0$ is the amplitude constant. Then, a larger amplitude of the negative imaginary part of $k_{\mathrm{rc}}$ (i.e., -Im$[k_{\mathrm{rc}}]$), indicates faster attenuation of the acoustic waves along the propagation direction, signifying the increased intrinsic loss. As shown in Figure S19, we have applied narrow region acoustic theory to calculate the changes in $k_{\mathrm{rc}}$ for a rectangular cavity with one side fixed at 100 mm, while the other side varies from relatively narrow to wide. Our findings demonstrate that as the cavity becomes narrower, -Im$[k_{\mathrm{rc}}]$ increases, leading to higher intrinsic losses. Similar conclusions can be drawn for

cavities with different cross-sectional shapes [57]. Consequently, the increased intrinsic loss results in lower quality factors and LDOS, according to the established theories presented in previous studies [46, 63].

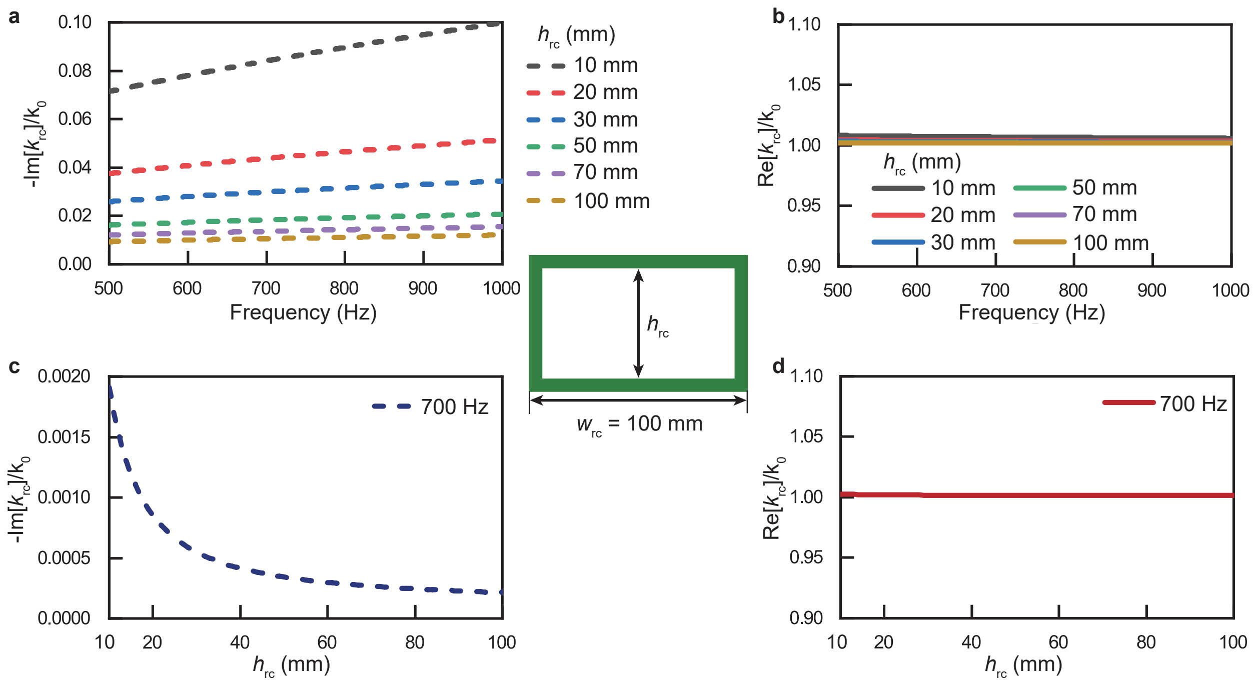


FIG. S19. Demonstration of the relationship between intrinsic losses and the structural narrowness of acoustic cavities. a-b) The imaginary parts and real parts of the normalized wave numbers with varying side widths ($h_{\mathrm{rc}}$). c-d) The imaginary parts and real parts of the normalized wave numbers with varying side widths at 700 Hz.
